# Targeting the Immune Microenvironment in the Treatment of Head and Neck Squamous Cell Carcinoma

**DOI:** 10.3389/fonc.2019.01084

**Published:** 2019-10-15

**Authors:** Hui-Ching Wang, Leong-Perng Chan, Shih-Feng Cho

**Affiliations:** ^1^Graduate Institute of Clinical Medicine, College of Medicine, Kaohsiung Medical University, Kaohsiung, Taiwan; ^2^Division of Hematology and Oncology, Department of Internal Medicine, Kaohsiung Medical University Hospital, Kaohsiung Medical University, Kaohsiung, Taiwan; ^3^Department of Otolaryngology-Head and Neck Surgery, Kaohsiung Medical University Hospital, Kaohsiung Medical University, Kaohsiung, Taiwan; ^4^Faculty of Medicine, College of Medicine, Kaohsiung Medical University, Kaohsiung, Taiwan

**Keywords:** head and neck cancer, microenvironment, biomarkers, immunotherapy, immunoresistance

## Abstract

Head and neck squamous cell carcinoma (HNSCC) is a highly aggressive solid tumor, with a 5-year mortality rate of ~50%. The development of immunotherapies has improved the survival of patients with HNSCC, but, the long-term prognosis of patients with recurrent or metastatic HNSCC remains poor. HNSCC is characterized by intratumoral infiltration of regulatory T cells, dysfunctional natural killer cells, an elevated Treg/CD8^+^ T cell ratio, and increased programmed cell death ligand 1 protein on tumor cells. This leads to an immunocompromised niche in favor of the proliferation and treatment resistance of cancer cells. To achieve an improved treatment response, several potential combination strategies, such as increasing the neoantigens for antigen presentation and therapeutic agents targeting components of the tumor microenvironment, have been explored and have shown promising results in preclinical studies. In addition, large-scale bioinformatic studies have also identified possible predictive biomarkers of HNSCC. As immunotherapy has shown survival benefits in recent HNSCC clinical trials, a comprehensive investigation of immune cells and immune-related factors/cytokines and the immune profiling of tumor cells during the development of HNSCC may provide more insights into the complex immune microenvironment and thus, facilitate the development of novel immunotherapeutic agents.

## Introduction

Head and neck cancer, 90% of which is squamous cell carcinoma (HNSCC), is the sixth most common cancer globally (1). HNSCC is composed of a heterogeneous group of tumors developing from the mucosa of the nasal and oral cavity, oropharynx, hypopharynx, or larynx (2). The major risk factors for HNSCC are smoking and alcohol consumption. Other risk factors include high risk human papillomavirus (HPV) infection, which is associated with oropharyngeal cancer increasingly worldwide (3). The areca nut chewing is linked to development of oral cancer in south Asia, Taiwan, and Pacific islanders. Treatment of HNSCC involves a multidisciplinary approach composed of surgery, radiotherapy, chemotherapy, and targeted therapy. However, the prognosis of metastatic HNSCC remains extremely poor. A combination of cetuximab and chemotherapy (cisplatin and 5-fluorouracil) shows better clinical efficacy than conventional chemotherapy; however, the median overall survival time is ~10 months (4). In recent years, the introduction of immune checkpoint inhibitors (ICIs) targeting the programmed death 1-programmed death ligand 1 (PD1-PDL1) pathway has resulted in further improvements in the outcome of patients with metastatic HNSCC, but the results remain unsatisfactory when compared with other malignancies, like melanoma and lung cancer (5, 6).

Accumulating data suggest that the tumor microenvironment (TME) plays an important role in the pathogenesis and development of treatment resistance in a variety of malignancies, including HNSCC. Several cell subtypes, including regulatory T cells (Tregs), cancer-associated fibroblasts, and macrophages, together with non-cellular components, like extracellular matrix (ECM), have been shown to be associated with immunocompromised status and the dysfunction of normal immune cells, like cytotoxic T cells or dendritic cells in the TME of HNSCC (7). HPV infection status and smoking are also related with distinct immune TMEs (8, 9).

To achieve improved treatment responses and clinical outcomes in the immunotherapy era, it is important to understand the complex immune TME of HNSCC. In this review, we describe major cell subtypes and cellular components and discuss their function. In addition, we summarize potential strategies to overcome TME-mediated treatment resistance.

## Tumor Microenvironment of HNSCC

The heterogeneity of molecular and cellular components has been reported in the TME of HNSCC (10, 11). However, the HNSCC TME is still characterized by some unique features, leading to immunosuppression and diminished anticancer immunity (Table 1). The TME is composed of stromal cells, immune cells, tumor cells, and cytokines, which mediate the interactions between these cells. HNSCC patients have decreased absolute T cell counts in the tumor and the circulation and the T cells have apoptotic features via the Fas/FasL signaling pathway and defective function (12, 16). The functional defects of tumor-infiltrating lymphocytes (TIL) include decreased expression of the CD3 zeta chain, decreased cytokine secretion, and loss of the ability to kill cancer cells (13–15). Tregs account for the major proportion of T cell components, which construct an immunosuppressive barrier, thus hindering the activity of effector T cells (Teffs) in the TME and interfering with the antitumor response to immunotherapy (26). A decrease in the number of immune cells with antigen-presenting machinery (such as dendritic cells) and in cytotoxic ability (such as natural killer cells) results in a profoundly immunodeficient tumor, which is common in HNSCC (16, 19, 27). Moreover, HNSCC tumors are characterized by desmoplastic stromal fibroblasts, which promote tumor invasion and progression via autocrine and paracrine factors (28, 29).

**Table 1:** Immune profilings of tumor microenvironment in HNSCC.

**Characteristics**	**Functions and mechanisms**	**References**
Decrease absolute T cell counts in tumor and circulation	Activation of Fas/FasL signaling pathway, leading to apoptosis of T cells	(12)
Dysregulation of T cell functions	1. Decreased HLA-DR expression on DCs and defective functions to stimulate allogeneic T cells 2. Decreased expression of the CD3 zeta chain (CD3ζ) 3. Decreased response to mitogens or IL-2 4. Absence of IL-2 and/or IFN-γ production	(13–15)
Downregulation of antigen processing machinery	Myeloid DCs is lower than lymphoid DCs	(16)
Increased Treg cell	1. Induce apoptosis of CD8^+^ T cells 2. Inhibition of the proliferation of CD4^+^ T cells	(12)
Increased MDSCs	Increased arginase-1 and iNOS driving immunosuppression partially by inactivating effector T cells	(17, 18)
Decreased NK cells	Impaired NK cell activity	(19)
Increased Activated, antigen-presenting and memory B cells		(20)
Increased expression of immune checkpoint ligand and receptors	A series of inhibitory immune checkpoints including PD-1, CTLA-4, TIM3, IDO, KIR, and TIGIT	(21–23)
Deficiencies or alterations of tumor HLA class I expression	Causing T-cell tolerance	(21)
Increased TGF-β, IL-6, and IL-10	Secreted by Tregs and MDSCs	(24, 25)
Aberrant activation of the transcription factors STAT3 and NF-kB	Related to IL-6 and TGF-β signaling, respectively	(24, 25)
Increase enzymes IDO-mediated degradation of the amino acid tryptophan	1. Deprivation of the tumor microenvironment of essential nutrients for T cell function 2. Activate Tregs to overcome immunogenic responses and promote tumorigenesis	(17, 18)

*HLA, human leukocyte antigen; DC, dendritic cells; IL, interleukin; IFN, Interferon; DC, dendritic cells; MDSC, myeloid-derived suppressor cells; iNOS, inducible nitric oxide synthase; PD-1, Programmed death-1; CTLA-4, cytotoxic T-lymphocyte–associated antigen 4; TIM3, T-cell immunoglobulin and mucin domain-3; IDO, indoleamine 23-dioxygenase; KIR, killer cell immunoglobulin-like receptors; TIGIT, T cell immunoreceptor with Ig and ITIM domains; TGF, transforming growth factor; STAT3, Signal transducer and activator of transcription 3; NF-kB, nuclear factor kappa light chain enhancer of activated B cells*.

Communication within cancer cells, immune cells, and stromal cells via extracellular vesicles (EVs) is increasingly thought to be important (30). EVs not only deliver oncogenic proteins and non-coding RNA molecules to modulate tumor progression, but also modulate immune responses by inhibiting T cell proliferation and Th1 and Th17 differentiation (31). EVs promote suppressive immunity by activating Fas ligand (FasL), to induce CD8^+^ T cell apoptosis and the polarization of THP-1 to tumor-associated macrophages (TAMs) of the M2 phenotype (32, 33). Although several studies have analyzed the TME, it remains difficult to define HNSCC as an immune-inflamed, immune-excluded, or immune-desert tumor, due to diverse intratumor/peritumor expression patterns and the distribution of immune cells and cytokines (34, 35). The antitumor immune response to immunotherapy in the TME depends on the balance of stromal components, intratumoral Teffs, and immune-suppressive cell populations.

### Cellular Component of the HNSCC TME

#### Regulatory T Cells

Tregs are a subset of T cells that contribute to the immunosuppressive TME in HNSCC (21). Treg recruitment is mediated by chemokines and associated receptors, such as CCL28-CCR10 and CXCL12-CXCR4 (36, 37). Tregs are characterized by specific markers, such as of CD4; CD25; and the transcription factor, forkhead box P3 (FOXP3) (22). Tregs express high levels of cytotoxic T-lymphocyte-associated protein 4 (CTLA-4), which binds to CD80 and CD86 on antigen-presenting cells (APCs), leading to a reduced capacity to activate Teffs. Tregs exhibit their suppressor function by the consumption of interleukin-2 (IL-2), the secretion of granzyme and/or perforin to damage effector cells, and the production of immune-inhibitory cytokines and molecules, such as IL-10, IL-35, and transforming growth factor-β (38, 39). Tregs release large amounts of ATP and provide inhibitory signals to Teffs and APCs via the engagement of adenosine A_2A_ receptor (A_2A_R) (40). In HNSCC, as in other malignancies, large numbers of Tregs infiltrate the TME. Intratumoral Tregs are more immunosuppressive than circulating Tregs, as evidenced by an increased expression of immune checkpoint molecules (23). A recent study identified a subset of Tregs with high levels of T-cell immunoglobulin and mucin domain-3 (TIM-3) expression from a population of CD4^+^CTLA-4^+^CD25^high^ Treg cells. These high TIM-3-expressing Tregs are more immunosuppressive than Tregs with low levels of TIM-3 expression. After the administration of an anti-PD-1 monoclonal antibody, the expression of TIM-3 on this subgroup of T cells decreased (41). Another recent study demonstrated that Tregs are related to resistance to radiotherapy. The incorporation of an anti-CD25 antibody can overcome Treg-related treatment resistance (42). Several studies have demonstrated a negative prognostic impact of large numbers of Tregs in HNSCC (43, 44).

#### Myeloid-Derived Suppressor Cells

Myeloid-derived suppressor cells (MDSCs) can be divided into three major subtypes, Ly6C^+^ monocytic MDSCs (M-MDSCs); Ly6G^+^ granulocytic polymorphonuclear myeloid-derived suppressor cells (PMN-MDSCs); and early stage e-MDSCs, which consist of the former two subsets deficient in myeloid lineage markers (45). The accumulation of MDSCs in the TME is associated with cancer progression and the inhibition of T cell activity and function (46). Various factors in the TME can induce the accumulation of MDSCs, including vascular endothelial growth factor (VEGF), IL-6, and granulocyte-macrophage colony-stimulating factor (GM-CSF) (20). In addition, MDSCs regulate the TME by increasing the production of nitric oxide, reactive oxygen species, inducible NO synthase, and arginase-1; depleting various amino acids, such as L-arginine, L-tryptophan, and L-cystein; inducing pro-angiogenic factors; and elevating the expression of PD-L1 (17, 18). In HNSCC, a recent study demonstrated that a higher frequency of PMN-MDSCs is associated with poorer survival. Specifically, a subset of CD66b^+^/CD11b^+^/CD16^+^ mature PMN-MDSCs showed higher expression and activity of arginase I and demonstrated a greater suppressing effect on T cell proliferation and cytokine production than other MDSC subtypes. Moreover, high levels of CD11b^+^/CD16^+^ PMN-MDSCs, but not other PMN-MDSC subsets, are strongly correlated with adverse outcomes in HNSCC patients (47).

#### Cancer-Associated Fibroblasts

Cancer-associated fibroblasts (CAFs) construct the stroma of the TME to promote the growth of cancer cells. CAFs possess different characteristics dependent on their status. For example, the active form of CAFs displays typical markers, such as α-smooth muscle actin and fibroblast activation protein and promotes tumor proliferation, invasion, and metastasis (48–50). CAFs regulate the TME via secretion of various cytokines and growth factors, such as VEGF, epidermal growth factor, C-X-C motif chemokine ligands, and C-C motif chemokine ligands (CCLs) (51, 52). Most importantly, CAFs secrete matrix-metalloproteinases (MMPs), which are crucial regulators of the TME and are responsible for degradation of the ECM (53). CAFs can be transformed from diverse progenitor cells, including endothelial cells, resting fibroblasts, and epithelial cells, via mesothelial-mesenchymal transition or epithelial-mesenchymal transition (EMT) (54, 55). In the TME of HNSCC, CAFs can promote the proliferation, migration, and invasion of tumor cells (29). CAFs also have a metabolic relationship with tumor cells. CAFs secrete hepatocyte growth factor (HGF), which then activates c-met to promote the progression of HNSCC (56). Additionally, HNSCCs secrete basic fibroblast growth factor (bFGF) which increases the phosphorylation of p44/42 mitogen-activated protein kinase, leading to the secretion of HGF from CAFs. Notably, the secretion of bFGF is also mediated by CAF-secreted HGF. Inhibition of c-met and the FGF receptor can reduce tumor volume. CAFs are also associated with the development of cancer stem cells, which is associated with treatment resistance (57). CAF secretes periostin, which promotes a cancer stem cell-like phenotype via interaction with protein tyrosine kinase 7 (58). Another study also showed that CAFs secrete several proteins that promote the expression of stemness-associated genes in HNSCC cells. Inhibition of these protein-associated pathways can suppress tumor growth (59).

#### Tumor-Associated Macrophages

TAMs have two distinct phenotypes, M1 and M2, with different morphological and biological characteristics (60, 61). The activated M1 phenotype promotes Th1 response and displays pro-inflammatory behaviors, whereas the activated M2 phenotype enhances Th2 response and mediates anti-inflammatory functions, which are more associated with tumor progression, invasion, metastasis, and the suppression of T cell immunity (61–63). Activated M2 macrophages demonstrate upregulated levels of IL-10, arginase-1, and peroxisome proliferator-activated receptor γ, which are known as markers of M2 TAMs (64–66). The M2 phenotype is induced by several cytokines, such as IL-4, IL-10, and IL-13. Activated M2 macrophages inhibit M1 TAMs and promote tissue remodeling through the production and secretion of anti-inflammatory cytokines, including IL-1 receptor antagonist, IL-10, transforming growth factor-β (TGF-β), VEGF, and tumor necrosis factor-α (TNF-α) (24, 25). In HNSCC, TAMs are recruited to the TME and directly contact SCC cells. A recent study showed that CCL18 derived from M2 macrophages is able to promote tumor metastasis by inducing EMT and stemness (67). Regarding clinical significance, a meta-analysis showed that high CD68^+^ and CD163^+^ TAM density is associated with poor cell differentiation and advanced disease status (68). Another meta-analysis showed that high stromal levels of CD163^+^ TAMs are associated with poorer overall and progression-free survival (69).

#### Other Cellular Subtypes

Human natural killer (NK) cells are important in the innate immune system and can be classified into two subgroups according to the surface expression of CD56 and CD16. CD56^dim^/CD16^bright^ NK cells are predominantly responsible for natural cytotoxicity, whereas CD56^bright^/CD16^dim^ NK cells regulate immune reactions through the secretion of cytokines, such as interferon-γ and TNF-α (70, 71). The activation of NK cells induces the apoptosis of target cancer cell, through the exocytosis of perforin and granzymes, FasL and TNF-related apoptosis-inducing ligand (TRAIL) activation, or antibody-dependent cellular cytotoxicity (ADCC) (72, 73). The natural killer group 2D (NKG2D) receptors on immune cells, including NK and several T cell subsets, play an important role in immunosurveillance. By identifying and engaging the NKG2D ligand (NKG2DL) on tumor cells, NK and T cells can exert anti-tumor effects. In HNSCC, high plasma levels of shed NKG2DLs correlate with NK cell inhibition and disease progression (74).

Neutrophils are involved in the adaptive immunity response. Tumor-associated neutrophils (TANs) exhibit both pro- and anti-tumor characteristics. Similar to TAMs, TANs are also divided into two subgroups, N1 and N2 (75). Neutrophils eradicate cancer cells by releasing the antimicrobial and cytotoxic contents of their granules or by secreting immune mediators to recruit other antitumor effector cells. However, other factors from the tumor can shift neutrophils into a pro-tumor phenotype (76). Neutrophils with the pro-tumor N2 phenotype possess CXCR4, VEGF, and MMP-9 markers, which facilitate tumorigenesis, promote tumor growth, stimulate angiogenesis, and mediate immunosuppression (75).

### Non-cellular Components in the TME

The ECM contains large composites of non-cellular factors, including structural proteins, growth factors, proteoglycans, and glycoproteins, which form the main structure of the TME (77). MMPs, which are mainly produced by the ECM, are a large family of proteins and peptide hydrolases that mediate the degradation of the ECM and facilitate the migration of cancer cells (78). MMPs also activate bFGF, VEGF, and TGF-β and promote angiogenesis (79, 80). Fibronectin is the major glycoprotein in the ECM and it plays a crucial role in interactions between other molecules, such as integrins, collagens, and fibrin (81, 82). Increased levels of fibronectin are associated with tumor invasion, progression, and resistance to treatment (83, 84). Other molecules are also involved in cell adhesion and proliferation and assist in supporting the surrounding TME.

### HPV Infection and Smoking Are Associated With a Distinct Immune TME

#### HPV Infection

HPV infection plays a pivotal role in the immune modulation of HNSCC. In general, HPV-positive HNSCCs demonstrate relatively inflamed immunity compared with HPV-negative HNSCCs (Table 2). A TME with a prolonged viral infection induces anti-tumor immunity via the expression of tumor-associated antigens (TAAs) and tumor-specific antigens in immune cells and tumor cells (8). After cytotoxic therapies (radiotherapy or chemotherapy), the antigen-processing machinery (APM) promotes the expression of major histocompatibility complex (MHC) class I molecules to present the antigen peptide from dying tumor cells to T cells (89). In addition, an increase in the infiltration of NK cells and T cells, including CD3^+^, CD4^+^, and CD8^+^ TILs, creates a vigorous TME that stimulates cellular immunity in HPV-positive HNSCCs (85, 86). Interestingly, HPV-positive oropharyngeal cancer demonstrates higher CD4^+^, higher CD8^+^, and lower CD4^+^/CD8^+^ ratio compared with HPV-negative HNSCC (85). Humoral immunity is also induced by the recruitment of CD19^+^/CD20^+^ B cells (87). Antigen presentation and cytotoxicity are promoted by gathering dendritic cells (DCs) and APCs (86). An increase in the number of intratumor and peritumor infiltrating immune cells results in a favorable prognosis and enhances the response to radiotherapy and immunotherapy (34). The interaction between HPV-negative oropharyngeal cancer cells and CAFs results in the secretion of chemokines via an IL-1/IL-1R-mediated mechanism, which is less prominent within the HPV-positive TME (88). Thus, the metabolic profiles are quite different between HPV-positive and HPV-negative HNSCCs.

**Table 2:** Different immune modulations between HPV-negative and HPV-positive HNSCC.

**HPV negative HNSCC**	**HPV positive HNSCC**	**References**
Lower CD3^+^ T cells	Higher CD3^+^ T cells	(85, 86)
Lower CD4^+^ T cells	Higher CD4^+^ T cells	(85, 86)
Lower CD8^+^ T cells	Higher CD8^+^ T cells	(85, 86)
Increased CD4^+^/ CD8^+^ ratio	Decreased CD4^+^/ CD8^+^ ratio	(85, 86)
Lower CD45^+^ cells, CD8^+^ cells, CD8^+^ IFNγ^+^ cells, and CD8^+^IL-17^+^ cells	Higher CD45^+^ cells, CD8^+^ cells, CD8^+^ IFNγ^+^ cells, and CD8^+^IL-17^+^ cells	(85, 86)
Lower CD45^+^ lymphocytes and CD19^+^/CD20^+^ B cells	Higher CD45^+^ lymphocytes and CD19^+^/CD20^+^ B cells	(87)
Higher Treg cells	Lower Treg cells	(85, 86)
Low CD56^dim^ NK cells	High CD56^dim^ NK cells	(85, 86)
Lower tumor-infiltrating APCs	higher tumor-infiltrating APCs	(86)
Lower myeloid and plasmacytoid DCs	Higher myeloid and plasmacytoid DCs	(86)
Lower DC signatures, including CD103, and CD11C	Higher DC signatures	(86)
Lower levels of chemokines	Higher levels of chemokines	(88)
Higher levels of Cox-2 and Tim-3 mRNA	Lower levels of Cox-2 and Tim-3 mRNA	(86)
Lower levels of PD-1 mRNA	Higher levels of PD-1 mRNA	(86)
Lower “T-cell exhaustion markers,” including LAG3, PD-1, TIGIT, TIM3, and CD39	Higher “T-cell exhaustion markers”	(87)
Lower levels of cytotoxic mediators, including granzyme A, granzyme B, and perforin	Higher levels of cytotoxic mediators	(87)
Exosomes suppressed DC maturation and expression of APM components	Exosomes promoted DC maturation and did not suppress expression of APM components in mature DCs	(89)
Increased MAGEA1 and MAGEA3 gene expression	Increased CDKN2A gene expression	(87)

*IFN, interferon; IL, interleukin; NK cells, natural killer cells; APC, antigen-presenting cell; DC, dendritic cells; Cox-2, cyclooxygenase-2; Tim-3, T-cell immunoglobulin and mucin domain-3; LAG-3, lymphocyte-activation gene 3; PD-1, Programmed death-1; TIGIT, T cell immunoreceptor with Ig and ITIM domains; APM, antigen processing machinery; MAGEA, melanoma-associated antigen; CDKN2A, cyclin-dependent kinase Inhibitor 2A*.

The communication vesicles, EVs, also display different features depending on viral status. In HPV-positive cancers, exosomes carry viral proteins, genes, and TAAs (90, 91). However, these differences in EVs do not occur by influencing the T cell response. The functions of both CD4^+^ and CD8^+^ T cells are suppressed by these exosomes. The expression of co-stimulatory CD80 and CD83 molecules on immature DCs is up-regulated, but the expression of APM components is not suppressed in HPV-positive exosomes. In contrast, HPV(–) exosomes inhibit DC maturation and APM component expression (8). Moreover, HPV-negative tumors have a more active metabolic signature, with elevated expression of genes associated with glycolysis and oxidative phosphorylation (92). HPV-negative tumors are also characterized by increased MCT1 expression, which indicates that the regulation of lactate homoeostasis is more significant in promoting the invasion of HPV-negative HNSCCs (93).

#### Smoking

Smoking is a risk factor for the development of HNSCC and it promotes pro-inflammatory and immunosuppressive effects, which impact the TME of HNSCCs, to facilitate tumor development (94, 95). Smoking results in enrichment of immunogenic neoantigens which cause both pro- and anti-immunity effects in smoking-associated cancers, including lung cancer and HNSCC. In lung cancer, smoking leads to increased neoantigens and constructs an inflamed TME, which suggests higher response rates to ICIs in smokers. In contrast, the enhancement of immunogenic neoantigens by smoking forms a more immunosuppressive in TME in HNSCC by increased T cell apoptosis which is mediated through reactive oxygen and nitrogen species (94). In the TCGA database, enrichment scores from two Gene Expression Omnibus cohorts were higher in never-smoker and never-drinker (NSND) patients compared with smoker and drinker (SD) patients. To identify biological differences, gene set enrichment analysis of the TCGA dataset was performed and immunity-associated pathways were found to predominantly involve T-cell activation and differentiation in NSND patients. The TME in NSND patients is more immunoactive than the TME of SD patients, including an increased number of CD8^+^ TIL cells; increased INF-γactivation; overexpression of immune checkpoint ligands and receptors, such as indoleamine 23-dioxygenase 1 (IDO1) and PD-L1; and higher scores in the pembrolizumab-response signature (96). Tobacco smoking attenuates the cytotoxicity of the TME by repressing CD8^+^ T cells, NK cells, and DCs (9). Overall, smoking has a negative impact on immune responses, regardless of alcohol consumption.

## Mechanisms of TME-Mediated Drug Resistance in HNSCC

The mechanisms of resistance to epidermal growth factor receptor (EGFR) inhibitors have been known for decades and they include nuclear localization of EGFR, activation of other ErbB family receptors, mutant forms of the receptor (EGFRvIII), or cross-talk with other signaling pathways (97, 98). However, issues of resistant mechanisms to immunotherapy have been gradually emphasized recently. These include a lack of production, editing, and presentation of neo-antigens; impaired intratumoral immune infiltration; impaired IFNγ signaling; immune factors within the TME; upregulation of alternative immune checkpoints; severe T-cell exhaustion; and T-cell epigenetic changes (99–102).

The downregulation of human leukocyte antigen (HLA) class I molecules and loss of β2-microglobulin expression interferes with antigen presentation to cytotoxic T cells (103). Specific oncogenic signaling pathways change the TME. Loss of the PTEN induces the expression of CCL2 and VEGF and blocks T-cell infiltration, leading to resistance to ICIs (104). Alterations in β-catenin/WNT signaling decrease CCL4 production and hinder the infiltration of DCs (105). During the development of ICI resistance, the TME shows an increase in the number of effector memory CD8 T cells (CCR7^−^CD45RA^−^), a lower CD4/CD8 ratio, and upregulation of TIM-3 on CD4 and CD8 T cells (100). Moreover, the major regulators of therapeutic response and resistance are Tregs and TAMs. In preclinical HNSCC mouse models, the Treg population is elevated during tumor rebound after combined treatment with ICI and radiation (26). Depletion of major histocompatibility complex class II-low TAMs increases chemotherapy-related DNA damage and apoptosis (106). Depletion of tumor-infiltrating Tregs using an anti-CD25 antibody, enhances the binding ability of activating Fc gamma receptors, increases Teff:Treg ratios, and improves the response to ICIs (107). High levels of alternative co-inhibitory receptors on T cells (e.g., CTLA-4, TIM-3, lymphocyte-activation gene 3, and V-domain Ig suppressor of T cell activation) and high levels of immune-suppressive cytokines or metabolites, causes T cell exhaustion, which also induces ICI resistance (108).

## Potential Strategies to Overcome TME-Mediated Drug Resistance

### Novel Therapeutic Agents or Combination Therapies

**Figure 1:**
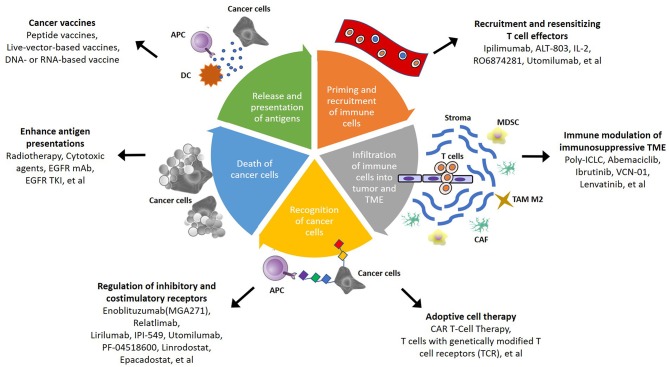
Schematic summary of potential strategies to overcome immunosuppressive TME in head and neck squamous cell carcinoma (HNSCC). In cancer-immunity cycle, there are several therapeutic strategies that can be applied to overcome TME-mediated treatment resistance. The steps of immune responses involve priming and recruitment of immune cells, infiltration of immune cells into tumor, and TME, recognition and death of cancer cells, then release and presentation of antigen from cancer cells. Targeting different mechanism of immune response become more potential therapeutic approach in the future.

Due to the insufficient response elicited by immunotherapy alone, several mechanisms for the regulation of immunoresistant niches have been proposed, including defective immunorecognition, tumor insensitivity to T cell effector molecules, an immunosuppressive TME, and the compensatory regulation of multiple inhibitory and costimulatory immune checkpoints (Figure 1) (109). Combinations of diverse agents targeting distinct mechanisms have been investigated in recent years (Table 3).

**Table 3:** Combination therapy to enhance PD-1/PD-L1-based treatment efficacy.

**Strategy**	**Treatments**	**Therapeutic modalities**	**Potential mechanisms**	**Phase**	**NCT ID status**
**Immunorecognition**					
Enhance antigen presentations	Radiotherapy	• RT (1 fraction, 8 Gy) + Pembrolizumab • RT (5 fractions, 4 Gy) + Pembrolizumab • Pembrolizumab + RT (1 fraction, 8 Gy) + Pembrolizumab • Pembrolizumab + RT (5 fractions, 4 Gy) + Pembrolizumab	1. Induce cell death to promote antigen presentation 2. Trigger activation of cGAS-STING pathway to enhance T-cell response 3. Adjust stromal TME	1	NCT02318771 Active, not recruiting
	Radiotherapy Cytotoxic agents	• Pembrolizumab + Cisplatin + RT • Placebo + Cisplatin + RT		3	NCT03040999 Active, not recruiting
	Radiotherapy Cytotoxic agents	• Docetaxel + Cisplatin + Nivolumab + Radioimmunotherapy		2	NCT03894891 Recruiting
	Radiotherapy CTLA-4 inhibitor	• Nivolumab + Ipilimumab + RT		1	NCT03162731 Recruiting
	Radiotherapy Cytotoxic agents EGFR mAb	• Nivolumab + Cisplatin • Nivolumab + High-dose Cisplatin • Nivolumab + Cetuximab • Nivolumab + IMRT		1	NCT02764593 Active, not recruiting
	EGFR mAb	• Pembrolizumab + Cetuximab	1. Stimulate antibody-dependent cell-mediated cytotoxicity 2. Prime adaptive and innate cellular immunity 3. Competitively inhibit the binding of EGF and other ligands (TGF-α)	2	NCT03082534 Recruiting
		• Avelumab + Cetuximab + RT		1	NCT02938273 Active, not recruiting
	EGFR TKI	• Nivolumab + Afatinib	1. Downregulate PD-L1 expression 2. Reduce PD-L1 expression via inhibiting NF-κB 3. Block the immune escape by upregulating the expression of NKG2D ligands on tumor cells and NKG2D on NK cells 4. Enhance the susceptibility to NK cell-mediated lysis by induction of ULBP1 by inhibition of PKC pathwy	1	NCT03652233 Withdrawn
		• Pembrolizumab + Afatinib		2	NCT03695510 Not yet recruiting
Resensitizing T cell effectors	Interleukin	• ALT-803 + Pembrolizumab • ALT-803 + Nivolumab • ALT-803 + Atezolizumab • ALT-803 + Avelumab	1. IL-15 superagonist 2. promote CD8^+^ T and NK cell expansion and function	2	NCT03228667 Recruiting
		• IL-2 + Pembrolizumab + Hypofractionated RT	1. Intralesional IL-2 2. increase PD-L1 expression and CD8^+^ T cell infiltration	1/2	NCT03474497 Recruiting
		• RO6874281+ Atezolizumab	1. IL-2 Variant (IL-2v), engineered IL2v moiety with abolished binding to IL-2Ra 2. targeting Fibroblast Activation Protein-A 3. Activation of immune effector CD8 T and NK cells, reduce activity on Tregs	2	NCT03386721 Recruiting
**Immune modulation of immunosuppressive TME**					
DC/NK cells	Carboxymethylcellulose, polyinosinic-polycytidylic acid, and poly-L-lysine dsRNA	• IV Durvalumab + IV Tremelimumab + IT/IM Poly-ICLC	1. Synthetic dsRNA complex which directly activate DCs and trigger NK cells to kill tumor cells 2. Induce interferon-γ production	1/2	NCT02643303 Recruiting
Cell cycles	CDK4/6 inhibitor	• Abemaciclib + Nivolumab	Create an immune inflamed TMEs through T cell activation and tumor cell intrinsic effects	1/2	NCT03655444 Recruiting
Cytokines	BTK inhibitor	• Ibrutinib + Nivolumab • Ibrutinib + Cetuximab	1. Inhibit IL-2 inducible T-cell kinase (ITK) 2. Maintain balance between Th1/Th2 T cells	2	NCT03646461 Recruiting
HU	Stroma	• VCN-01 and Durvalumab	Tumor-selective replication-competent adenovirus expressing PH20 hyaluronidase	1	NCT03799744 Recruiting
VEGF	VEGF	• Lenvatinib + Pembrolizumab	1. Reduce tumor associated macrophages 2. Enhance the ratio of memory T cells	1b/2	NCT02501096 Recruiting
**Regulation of inhibitory and costimulatory receptors**					
Inhibitory receptor	B7-H3 (CD276)	• Enoblituzumab (MGA271) + Pembrolizumab	1. Synergistic antitumor activity 2. Engagement of both innate and adaptive immunity 3. Modulation of T-cell immunosuppression 4. Decrease the risk of auto-immune related AE	1	NCT02475213 Active, not recruiting
	LAG-3	• Relatlimab • Relatlimab + Nivolumab	1. Synergistic antitumor activity 2. Positively regulate effector T cell function	1/2	NCT01968109 Recruiting
	KIR	• Nivolumab • Nivolumab + Lirilumab • Nivolumab + Ipilimumab + Lirilumab	1. Block interaction between KIR2DL-1,-2,-3 inhibitory receptors and ligands 2. Promote effector T cell function 3. Reverse T cell exhaustion	1/2	NCT01714739 Active, not recruiting
	PI3K	• IPI-549 and Nivolumab	Transform macrophages from an immune-suppressive to an immune-activating phenotype	1	NCT02637531 Recruiting
	CTLA-4	• Nivolumab with Ipilimumab • Nivolumab	1. CTLA-4 inhibitor: induce a proliferative signature in a subset of memory T-cells 2. PD-1 inhibitor: modulate genes that are involved in T-cell or NK-cell effector functions 3. Increase in plasma cytokine or chemokine levels	2	NCT02919683 Recruiting
		• Nivolumab and Ipilimumab • Nivolumab and placebo		2	NCT02823574 Active, not recruiting
Stimulatory receptor	4-1BB (CD137) OX40 TLR9 agonist	• Cohort A5: Avelumab + Utomilumab (Human IgG2 4-1BB mAb) • Cohort F1: CMP-001(VLP-encapsulated TLR9 agonist) +Avelumab • Cohort F2: CMP-001 + Avelumab + Utomilumab • Cohort F3: CMP-001 + Avelumab+PF-04518600 (OX40 agonist)	1. Utomilumab: production of IFN-γ and IL-2; stimulate and increase NK cells and T cells 2. PF-04518600: co-stimulate effector T cells and deplete regulatory T cells, resulting in enhanced tumor immunity 3. CMP-001: release the oligonucleotide into APCs	1	NCT02554812 Recruiting
	4-1BB (CD137)	• PF-04518600 • PF-04518600 + Utomilumab (PF-05082566)		1	NCT02315066 Active, not recruiting
Other pathway	IDO1	• Nivolumab and Linrodostat (BMS986205) • Nivolumab	1. Inhibitor of indoleamine 2,3-dioxygenase 1, a cytosolic enzyme for oxidation of tryptophan into kynurenine. 2. Inhibition of IDO1–kynurenine–AhR signaling	2	NCT03854032 Recruiting
		• Nivolumab + Linrodostat • Cetuximab + Cisplatin/Carboplatin + Fluorouracil		3	NCT03386838 Withdrawn
		• Nivolumab + Epacadostat • Nivolumab + Epacadostat + Chemotherapy		1/2	NCT02327078 Active, not recruiting
		• Pembrolizumab + Epacadostat • Pembrolizumab • EXTREME regimen		3	NCT03358472 Active, not recruiting

*RT, radiotherapy; Gy, gray; TME, tumor microenvironment; CTLA-4, cytotoxic T-lymphocyte–associated antigen 4; EGFR, epidermal growth factor receptor; mAb, monoclonal antibody; IMRT, intensity-modulated radiotherapy; EGF, epidermal growth factor; TGF, transforming growth factor; TKI, tyrosine kinase inhibitor; PD-L1, Programmed death-ligand 1; NK cells, Natural killer cells; IL, interleukin; DC, dendritic cells; IV, intravenous; IT, intratumoral; IM, intramuscular; dsRNA, double-stranded RNA; BTK, Bruton's tyrosine kinase; Th cells, T helper cells; HU, hyaluronidase; VEGF, vascular endothelial growth factor; AE, adverse event; LAG-3, lymphocyte-activation gene 3; KIR, killer cell immunoglobulin-like receptors; PI3K, phosphoinositide 3-kinases; IFN, Interferon; TLR9, Toll-like receptor 9; VLP, virus-like particle; APC, antigen-presenting cell; IDO1, indoleamine 23-dioxygenase 1; AhR, aryl hydrocarbon receptor*.

Defective immunorecognition involves dysfunction of antigen presentation in tumor cells, anergy of tumor-specific cytotoxic T lymphocyte, and immunoediting. Radiotherapy and cytotoxic therapy (NCT02318771, NCT03040999, NCT03894891, NCT03162731, NCT02764593, NCT02938273) induce cell death to promote antigen presentation and trigger activation of the cGAS-STING pathway to enhance the T-cell response. Moreover, radiation adjusts the stromal TME (110). Cetuximab (NCT02764593, NCT03082534, NCT02938273) binds to EGFR and to the CD16 receptor on NK cells and DCs, resulting in innate and adaptive immune responses, including ADCC and T cell priming (111). Afatinib (NCT03652233, NCT03695510), an EGFR tyrosine kinase inhibitor, downregulates PD-L1 expression via the inhibition of NF-κB. However, afatinib hinders immune escape by increasing the expression of NKG2D ligands on tumor cells and NKG2D on NK cells (112). ALT-803 (NCT03228667), an IL-15 superagonist, promotes CD8^+^ T cell and NK cell expansion and function and has demonstrated anti-tumor efficacy in preclinical models (113). Intralesional IL-2 (NCT03474497) increases PD-L1 expression and promotes CD8^+^ T cell infiltration (114). RO6874281 (NCT03386721), an engineered IL2v moiety, maintains its affinity for IL-2Rβγ, thus activating effector CD8 T cells and NK cells and reducing Treg activity (115).

The immunosuppressive TME also contributes to the low sensitivity of HNSCC to ICIs. Modulating different components of the TME improves the efficacy of ICIs and enhances self-immunity. Poly-ICLC (NCT02643303), a carboxymethylcellulose, polyinosinic-polycytidylic acid, and poly-L-lysine dsRNA, is a synthetic dsRNA complex that directly activates DCs, triggers NK cells, and induces interferon-γ production (116). Abemaciclib (NCT03655444), a CDK4/6 inhibitor, creates an immune inflamed TME through T cell activation and intrinsic tumor cell effects (117). Ibrutinib (NCT03646461), a Bruton's tyrosine kinase inhibitor, inhibits IL-2 inducible T-cell kinase (ITK), to strengthen specific anti-tumor responses (118). ITK plays a crucial role in maintaining the balance between Th1 and Th2 T cells. VCN-01 (NCT03799744), a selective oncolytic adenovirus encoding the human glycosylphosphatidylinositol-anchored enzyme, PH20 hyaluronidase, shows potential anti-tumor effects. Replication of the injected adenovirus in tumor cells results in cell death and the infection of adjacent tumor cells. Hyaluronidase also degrades hyaluronic acid (HA), which is abundant in the ECM and inhibits tumor cell growth and metastasis (119). Lenvatinib (NCT02501096), a multikinase inhibitor of VEGFR 1–3, fibroblast growth factor receptors (FGFR) 1–4, platelet-derived growth factor α receptors, RET, and KIT, reduces the number of TAMs and increases the ratio of memory T cells (120).

The regulation of inhibitory and costimulatory receptors synergically enhances the immunological anti-tumor effect. Inhibitory receptors, including B7-H3, LAG-3, killer cell immunoglobulin-like receptors (KIRs), phosphoinositide 3-kinases (PI3Ks), and CTLA-4, are applied in combination therapies. Enoblituzumab (NCT02475213), an Fc optimized, humanized IgG1 monoclonal antibody, promotes binding to activating FcγR and recognizes B7-H3, which is highly expressed in HNSCC. Combination therapy may contribute to synergistic antitumor activity (121). Relatlimab (NCT01968109), an anti-LAG-3 monoclonal antibody, shows an additive antitumor effect when administered with ICIs. LAG3 negatively regulates Teff function and is a marker of T cell exhaustion (122). IPI-549 (NCT02637531), a selective PI3K-γ inhibitor, transforms macrophages from an immune-suppressive to an immune-activating phenotype, which may help overcome resistance to ICIs (123). The well-known dual blockade therapy consisting of anti-CTLA-4 and anti-PD-1 antibodies (NCT02919683, NCT02823574), stimulates distinct immune cells and results in an inflammatory TME to overcome cancer cells (124). Similarly, cooperation with stimulatory receptors increases clinical benefits and treatment efficacy. Utomilumab (NCT01307267, NCT02315066), a 4-1BB/CD137 agonist, stimulates the activity and number of NK cells and T cells (125). PF-04518600 (NCT01307267, NCT02315066), a selective anti-OX40 antibody, activates OX40 and increases the proliferation of memory and effector T-lymphocytes (126). CMP-001 (NCT01307267), a Toll-like receptor 9 (TLR9) agonist, comprises a CpG-A oligodeoxynucleotide packaged in particles. It activates tumor-associated plasmacytoid DCs, which construct an interferon-rich TME and results in anti-tumor CD8^+^ T cell responses (127). IDO1, a major enzyme in tryptophan catabolism, is a target in clinical development, in combination with PD-1 ICIs (NCT03854032, NCT03386838, NCT02327078, NCT03358472). IDO1 converts tryptophan to kynurenine, which then activates aryl hydrocarbon receptor (AhR), a ligand-activated transcription factor, in Tregs, DCs, and NK cells. Activation of AhR induces subsequent cascades in three different cell types. In Tregs, AhR results in the nuclear translocation and enhancement of FoxP3 transcripts and IL10, eventually increasing Treg populations. In DCs, AhR promotes the production of IL-10 and inhibits IFNβ signaling. In NK cells, AhR induces the production of both IL-10 and IFNγ. The IDO1-kynurenine-AhR axis demonstrates a positive feedback loop. These effects on immune cells help establish an immunosuppressive TME. The inhibition of IDO reverses immunosuppression and enhances the response to ICIs (128).

### Cancer Vaccines

Cancer vaccines targeting HPV antigens and tumor-associated antigens enhance the immune response in HNSCC. Therapeutic vaccines include peptide vaccines, live-vector-based vaccines, and DNA- or RNA-based vaccines. Peptide vaccines derived from HPV antigens are taken up by DCs and displayed by either MHC class I, class II, or both molecules, after which they induce a T-cell mediated immune response. Several trials have investigated such drugs, including DPX-E7 (NCT02865135), GL-0817/GL-0810 (NCT00257738), P16_37-63 peptide (NCT01462838, NCT02526316), and ISA 101(NCT02426892). However, the response rates have been variable in relatively small populations of patients (129, 130). Live-vector-based vaccines are more immunogenic and induce strong pathogen-derived CD8 epitopes (131). Recent cancer vaccine modalities include DNA and RNA vaccines encoding selected tumor antigens or synthetic long peptide (SLP) vaccines co-delivering CD4 and CD8 epitopes (132). DNA or peptide vaccines targeting HPV E6 and E7 oncoproteins have demonstrated specific clinical efficacy in precancerous lesions and have shown promise in the treatment of HPV-related HNSCC. However, the development of vaccines against HPV-independent HNSCC has been less successful due to the difficulty in identifying available targets (133). Additional vaccine modalities are required to overcome the immunosuppressive TME in HNSCC.

### Cell-Based Therapy

T cells, including TILs, T cells with genetically modified T cell receptors (TCRs), and T cells transfected with chimeric antigen receptors (CAR), are the main types of cell-based therapy (134). Sufficient numbers of TILs overcome the immunosuppressive TME by removing other exhausted immune cells and inhibitory factors, such as cytokines. Adoptive immunotherapy using CAR T cells has displayed promising outcomes in hematological malignancies, such as leukemia and multiple myeloma. The process of CAR T cell therapy includes retrieving T cells from the patient's blood or tumor, training and stimulating their expansion in an *in vitro* system, and injecting the expanded cells back into the patient to promote cancer elimination. The development of tumor antigen-specific TCRs, for example HPV-targeted TCRs in genetically modify T cells, is another approach for adoptive immunotherapy. These modified T cells possess high levels of immune-signaling initiators and show rapid recognition of intracellular antigens, which can initiate an immune response against cancer cells. A phase I/II trial targeting the HLA-A^*^02:01-restricted epitope of E6 (E6 TCR T cells) enrolled patients with HPV-positive and HLA-A^*^02:01-positive metastatic epithelial cancers and showed that a dose up to 2 × 10^11^ cells was safe for patients. Partial responses in 2 of 12 patients (both with anal cancer) were reported (135). A phase I trial of T4 CAR T cell immunotherapy in HNSCC demonstrated safe intratumoral administration of T4 T-cells that co-express: (i) T1E28ζ, a CAR containing an ErbB ligand coupled to a CD28^+^CD3ζ endodomain and (ii) 4αβ, an IL-4-responsive chimeric cytokine receptor. Although a lymphopenia rate of 62% was observed, T4 manufacture was successful in 13/13 cases, yielding 2.5–7.5 Bn T cells (69 ± 13% transduced) (136). However, the development of adoptive cell therapy for HNSCC is still immature. There are still numerous difficulties and challenges including the identification of more specific peptide and genetic profiles of HNSCC cells. More precise knowledge of intracellular and extracellular neoantigens would help to identify potentially novel targets for cell therapy in HNSCC.

## Potential Biomarkers in HNSCC Immunotherapy

Potential biomarkers in HNSCC have been discussed for many years, but there is still no consensus. Recent studies have tended to focus on specific biomarkers, including PD-L1 expression, HPV status, tumor immune infiltration, immune-associated signatures, gene expression profiles (GEPs), tumor mutational burden (TMB), the status of DNA mismatch repair, and smoking-related signatures. PD-L1 immunohistochemistry is the most frequently used marker in clinical practice. However, there are several challenges in the clinical application of these biomarkers. For example, PD-L1 is a heterogeneous marker with different intratumoral/temporal and primary/metastatic variations in expression (137). Different immunohistochemistry assays have been used, with different thresholds for positivity and different scoring criteria, including a tumor proportional score (TPS) and a combined proportional score (CPS) (138). HPV status also influences immunity within the TME and affects responses to immunotherapy (6). TILs, defined as CD8^+^ T cells and Tregs, have demonstrated a possible role in distinguishing ICI responders from ICI non-responders (139). GEP and TMB, analyzed by microarray or next-generation sequencing platforms, have been investigated as predictive biomarkers for biological phenotypes and clinical outcomes in HNSCC. Some analyses have shown that TMB, CPS, and GEP can serve as independent predictive biomarkers for responsiveness to anti-PD-1/PD-L1 antibodies (140). Tumors with more mutations influencing the DNA damage response, for example those with mismatch repair deficiency (dMMR), have a higher TMB and are more sensitive to ICIs. This contributed to the FDA approval of pembrolizumab for patients with dMMR or MSI-H tumors, regardless of histology (141–143). Overall, while the interactions between the tumor, the immune system, and the microenvironment are complex, more reliable predictive biomarkers are required to assess tumor responsiveness to immunotherapy.

## Perspectives and Conclusions

As ICI monotherapy shows a durable response in only a small subset of patients, combination therapy with anti-PD-1/PD-L1 antibodies has emerged as an alternative and has shown encouraging results in the treatment of HNSCC. In addition, the anti-tumor effects of ICIs can be reinforced by increasing antigen presentation via radiation or chemotherapy/target therapy, modulating TME, or collaborating with costimulatory and inhibitory receptors on tumor cells or immune cells. The niches around cancer cells are crucial for interference with the efficacy of checkpoint inhibitors and they determine whether a tumor is “immunoactive” or “immunosuppressive.” Methods to overcome the immunotherapy resistance of the TME will become more crucial in the future. Multimodalities of treatment strategies aid in strengthening immunosurveillance and immunoediting. Studies to identify more specific targets for adoptive T cell therapies are ongoing. In addition, further studies designed to identify ideal biomarkers of individual tumors and to elucidate the mechanisms of immune escape are warranted.

## Author Contributions

H-CW, L-PC, and S-FC substantially contributed to the conception, drafting, editing, and final approval of this manuscript.

### Conflict of Interest

The authors declare that the research was conducted in the absence of any commercial or financial relationships that could be construed as a potential conflict of interest.
